# Trends in Dietary Patterns, Alcohol Intake, Tobacco Smoking, and Colorectal Cancer in Polish Population in 1960–2008

**DOI:** 10.1155/2013/183204

**Published:** 2013-11-28

**Authors:** Mirosław Jarosz, Włodzimierz Sekuła, Ewa Rychlik

**Affiliations:** Department of Nutrition and Dietetics with Clinic of Metabolic Diseases and Gastroenterology National Food and Nutrition Institute, Powsińska St. 61/63, 02-903 Warsaw, Poland

## Abstract

The study examined the relationships between long-term trends in food consumption, alcohol intake, tobacco smoking, and colorectal cancer (CRC) incidence. Data on CRC incidence rates were derived from the National Cancer Registry, on food consumption from the national food balance sheets; data on alcohol and tobacco smoking reflected official statistics of the Central Statistical Office. It was shown that CRC incidence rates were increasing between 1960 and 1995, which could have been affected by adverse dietary patterns (growing consumption of edible fats, especially animal fats, sugar, red meat, and declining fibre and folate intake), high alcohol consumption, and frequent tobacco smoking noted until the end of the 1980s. Since 1990, the dietary pattern changed favourably (decrease in consumption of red meat, animal fats, and sugar, higher vitamin D intake, increase in vegetables and fruit quantities consumed, and decline in tobacco smoking). These changes could contribute to the stabilisation of CRC incidence among women seen after 1996 and a reduction in the rate of increase among men.

## 1. Introduction

Colorectal cancer (CRC) is one of the main causes of cancer incidence among both genders worldwide, resulting from growing trend accounts now for approximately 9% of total cancers worldwide [1]. The World Cancer Research Fund/American Institute for Cancer Research report summarizes recent evidence which suggests that poor diet, body fatness, abdominal fatness, and physical inactivity are important modifiable risk factors for colorectal cancer.

**Figure 1:**
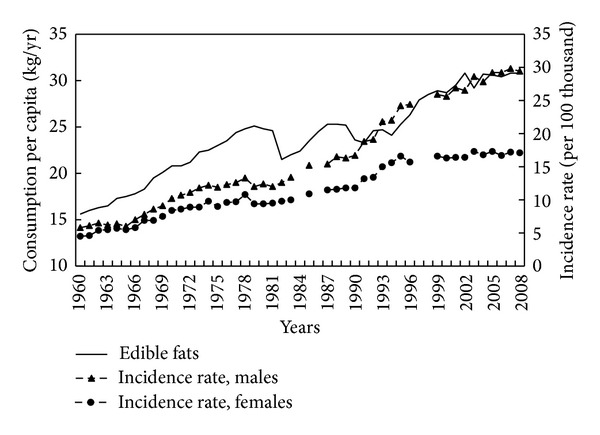
Edible fats consumption and colorectal cancer morbidity 1960–2008.

Colorectal cancer is one of the most common malignant tumours in Poland, particularly in men. In 2008, the age standardised incidence rate was 29.4/100,000 and 17.1/100,000 for men and women, respectively [2]. In contrast, rates in the end of the 1950s were much lower, amounting to 5.8/100,000 for men and 4.5/100,000 for women, respectively, in 1960 [3]. For many years there was an upward trend in CRC incidence, but the rate of growth for women was much lower in relation to men (see Figure 1). In consequence, incidence rates are now much more favourable in women (only 58% of the rate in men in 2008 compared with 78% in 1969), but there has been only small decline in this rate in women since 1996. The rate of growth in men slowed down from 1996 onwards, but it is only in the last 4 years covered by this analysis (2005–2008) that the trend may have levelled off.

The epidemiological evidence suggests several hypotheses on the association between diet and CRC risk [4]. The contribution of diet to cancer risk, prevention, and treatment has been a major subject of research in recent years due to the suggested protective role of some nutrients. Experimental studies suggest that specific dietary factors may influence growth, development, and differentiation of colorectal epithelial cells. For example, vegetables contain a large number of substances, both micronutrients with antioxidant activity, such as carotenoids and ascorbate, and other bioactive compounds with a variety of potent anticarcinogenic properties such as phenols, flavonoids, isothiocyanates, and indoles [1]. Animal model and human ecological epidemiological studies have suggested that selenium may be for adenomatous polypus and colon cancer [1]. A large intervention study demonstrated a modest but significant reduction of adenoma recurrence from calcium supplementation. Vitamin D and calcium together may have even more effect than either alone [5].

The impact of diet may in part be mediated by insulin sensitivity and inflammation [6], for which useful biomarkers exist. Studies suggest a positive relationship between high glycaemic index (GI) or glycaemic load (GL) diets and biomarkers of hyperinsulinemia, type 2 diabetes, and colorectal cancer, particularly among men. Insulin resistance (IR) and inflammation may act synergistically to promote CRC.

In the joint WHO and FAO report from 2003 experts have concluded that there is sufficient evidence that overweight and obesity increases the risk of colorectal cancer [7]. The review from 2008 indicated that in most studies body weight and BMI have been found to be positively related to risk of colon cancer in men, but weaker or no associations have been reported for women. However among the studies that have examined associations with rectal cancer, most have found no association with body weight or BMI [8]. The update from 2013 taking into account progress of the last five years regarding that issue concluded that colorectal cancer is one of the cancer sites that is undoubtedly linked to excess body fatness, rectal cancer is less affected by body fatness than colon cancer, and the risk relation with body fatness is stronger in men than in women [9].

Obesity, particularly abdominal obesity, has been associated with a state of chronic, low grade inflammation with persistent activation of nuclear transcription factor NF-*κ*B with subsequent transcription of genes that promote tumourigenesis [10]. It has been estimated that the relative risk (RR) of developing colorectal cancer in obese men and women is 1.24–1.59 and 1.09–1.22, respectively [10] compared with normal weight men and women.

Increased consumption of energy-dense, nutrient-poor foods with high levels of sugar and saturated fats, combined with reduced physical activity, have led to obesity rates that have risen threefold or more since 1980 in some areas of North America, the UK, Europe, the Middle East, Australasia, and China. An elevated body mass index has been associated with both the development of colonic adenomas and colorectal cancer [11].

There is also evidence that tobacco [12] and alcohol [13, 14] are also potential influences on CRC risk.

Repeat cross-sectional data on average food and nutrient intake in Poland since 1960, together with tobacco use and alcohol consumption, provide a useful basis for an ecological assessment of the possible impact of diet, tobacco, and alcohol on colorectal cancer incidence rates. Mentioned factors could influence spontaneous, nongenetic CRC, which accounts for the greater part of tumour burden. In our study we focus on correlations between incidence rates and selected factors as a way for looking at potential causation.

## 2. Materials and Methods

The study focused on assessment of the relationships between CRC incidence rates and consumption of edible fats, animal fats, red meat, sugar (excluding sugar going to the brewing industry) and the intake of dietary fibre, folate, calcium, and vitamin D. (“Consumption” refers to the average availability of food and nutrient based on national availability statistics (Food Balance Sheets), not the actual average amounts eaten.) Also, the potential influence of alcohol intake and tobacco smoking on CRC incidence was analysed using the same approach.

Data on CRC incidence rates were derived from the National Cancer Registry administrated by the Maria Skłodowska-Curie Memorial Cancer Center and Institute of Oncology in Warsaw [2, 3]. They include statistics for the following ICD codes: C18: colon, C19: rectosigmoid junction, and C20: rectum showing standardised incidence rates for men and women, covering the years between 1960 and 2008, except for 1984, 1986, 1997, and 1998, for which no data were available. The data represent in fact “registered incidence” and may be biased by some under registration [15]. The “standard world population” was applied as standard population to estimate standardised incidence rates [16].

Food availability over the same time period was determined using the database established by the National Food and Nutritional Institute [17–19]. This database covers data derived from the national food balance sheets. It shows quantities for a dozen or so major food groups available for consumption (expressed per capita/year). These amounts were converted into energy and nutrients intake (per capita/day) using national food composition tables which provide estimates of the energy and nutrient contents of foods (raw or as purchased, as appropriate) [20]. Dietary fibre content shown in the tables reflect total amount of soluble and insoluble fractions determined with the use of an AOAC (enzymatic-gravimetric) method [21].

Information on alcohol consumption (total expressed as litres of pure alcohol per annum, reflecting consumption of all major types of alcoholic beverages and their alcohol content) was derived from national statistical yearbooks [22]. In view of the fact that in Poland “vast majority of smokers (94.7%) smoke manufactured cigarettes” annual data on cigarette number smoked per capita were used [23].

The relationship between the above risk factors and CRC incidence rates were usually analyzed for the whole period of 1960–2008. However, due to character of the changes in the dietary pattern in the periods before and after the economic transformation, some analyses have been divided into two subperiods: 1960–1989 and 1990–2008.

Spearman's rank correlation coefficients were used as a measure of the relationship between colorectal cancer incidence rates and the selected parameters.

## 3. Results

Strong positive correlation between CRC incidence rates for men and women was found (0.99; *P* < 0.001).

**Figure 2:**
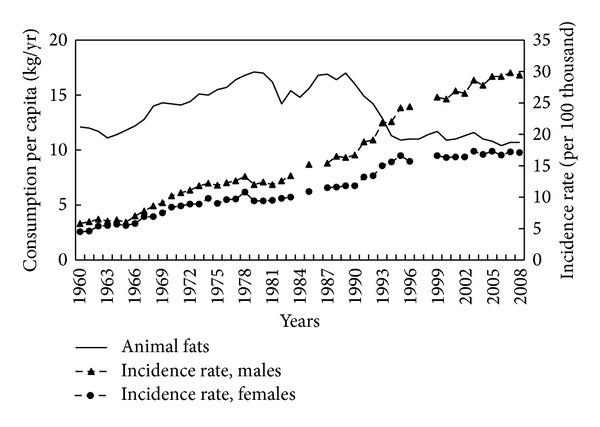
Animal fats consumption and colorectal cancer morbidity 1960–2008.

One of the dietary factors possibly responsible for the increase of CRC risk is fat consumption, especially animal fat. The years 1960–2008 saw a nearly twofold increase in the consumption of edible fats (Figure 1). Correlation coefficients between the consumption of this food group and the CRC incidence point to a significant relationship. A strong positive correlation has been demonstrated in this regard (0.93 for men and 0.92 for women; *P* < 0.001) (Table 1). In the case of animal fats, consumption in the subperiod covering years 1960–1989 showed a very high, positive correlation with incidence rates (0.86 for men and 0.84 for women; *P* < 0.001), when the share of animal fats in Poles' diet was very high (Figure 2). In the following years, the consumption of such fats declined significantly.

**Figure 3:**
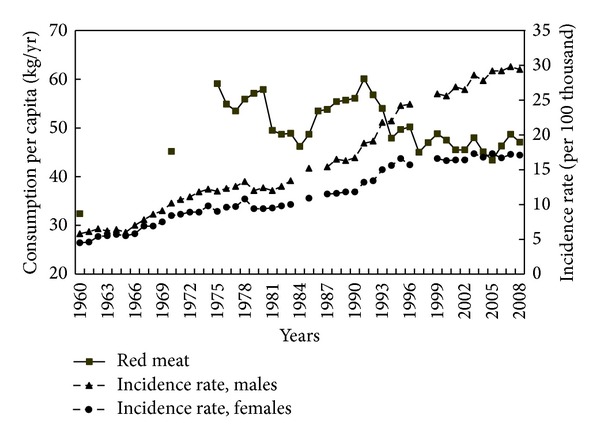
Red meat consumption and colorectal cancer morbidity 1960–2008.

A weaker correlation was found between red meat consumption and CRC incidence (−0.41, both genders). However, it may be assumed that the consumption of products from this group may have had a potential impact on morbidity. Data from years 1960–1975, though incomplete, indicate that the share of red meat in Poles' diet increased significantly (Figure 3). After this period, significant fluctuations were seen.

**Figure 4:**
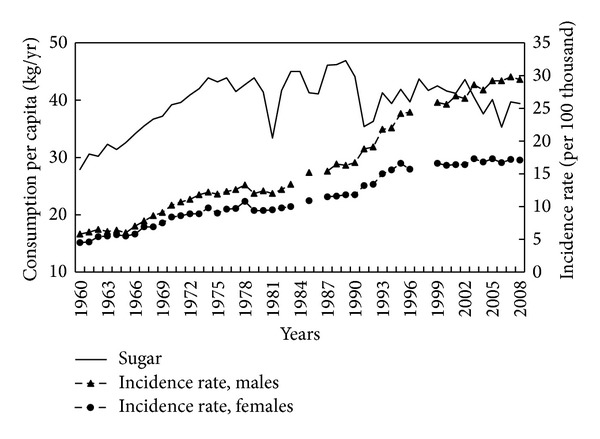
Sugar consumption and colorectal cancer morbidity 1960–2008.

In the years 1960–1989, sugar consumption increased by over 50% (mostly in the period 1960–1974) (Figure 4). During the period 1960–1989, there was a very high correlation between sugar consumption and CRC incidence rate (0.88 for men and 0.89 for women; *P* < 0.001). In the years from 1991 onward, sugar consumption showed a gradual (if fluctuating) decrease from its high point in the mid-1970s and 1980s.

**Figure 5:**
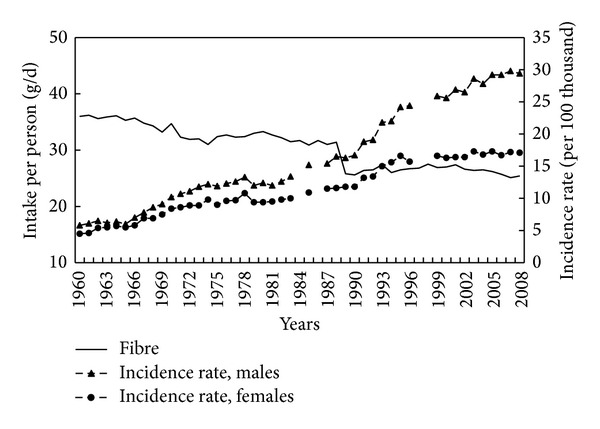
Fibre intake and colorectal cancer morbidity 1960–2008.

The level of dietary fibre, a potentially protective factors against CRC, fell from around 36 g/person/d in 1960 to around 25 g/person/d in 1989, since when the level has remained relatively constant (Figure 5). A strong negative correlation was observed between dietary fibre intake and incidence rates (−0.92 both for men and women; *P* < 0.001).

**Figure 6:**
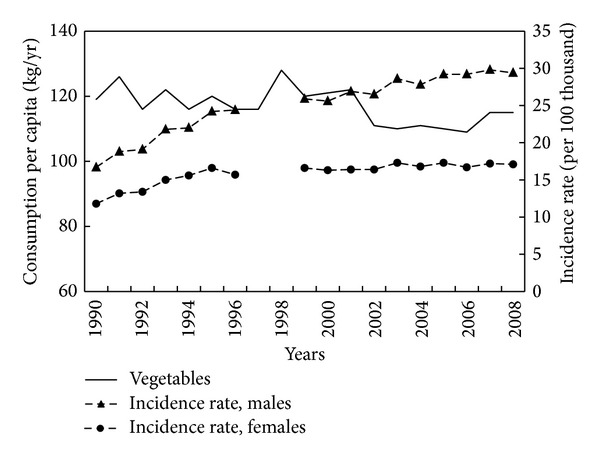
Vegetables consumption and colorectal cancer morbidity 1990–2008.

**Figure 7:**
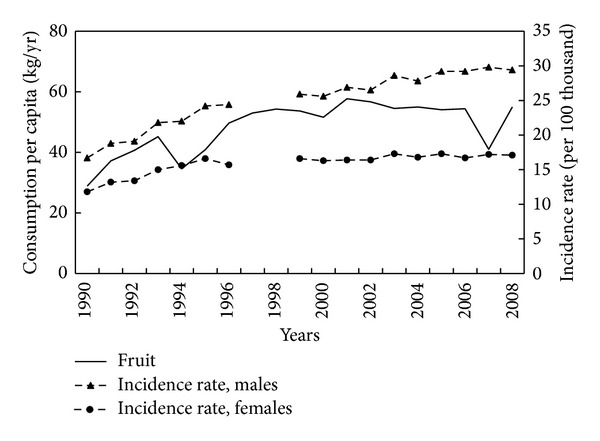
Fruit consumption and colorectal cancer morbidity 1990–2008.

Total vegetable and fruit consumption is potentially protective against CRC. Reported vegetable and fruit consumption fluctuated due predominantly to fluctuations in domestic production between 1960 and 1989, but from 1990 onwards showed more stable trends. The correlation of CRC incidence rates 1990–2008 with vegetable consumption was −0.66 in men and −0.67 in women (Figure 6), but with fruit consumption was unexpectedly positive, 0.70 in men and 0.62 in women (Figure 7).

**Figure 8:**
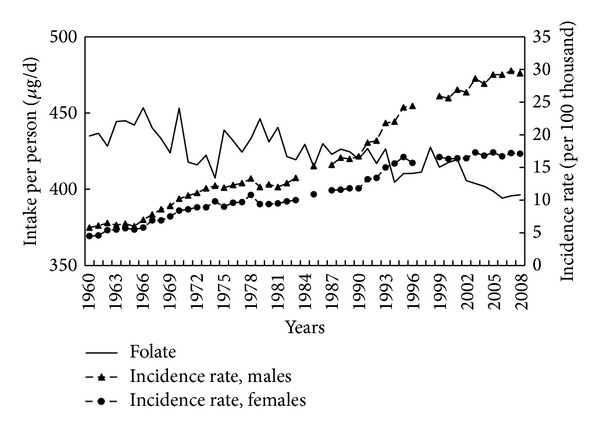
Folate intake and colorectal cancer morbidity 1960–2008.

Although there appear to have been marked fluctuations in folate intake between 1960 and 2008, the overall consumption has been decreasing (Figure 8). The colorectal cancer incidence rate showed a very high negative correlation (−0.53) with this vitamin intake.

**Figure 9:**
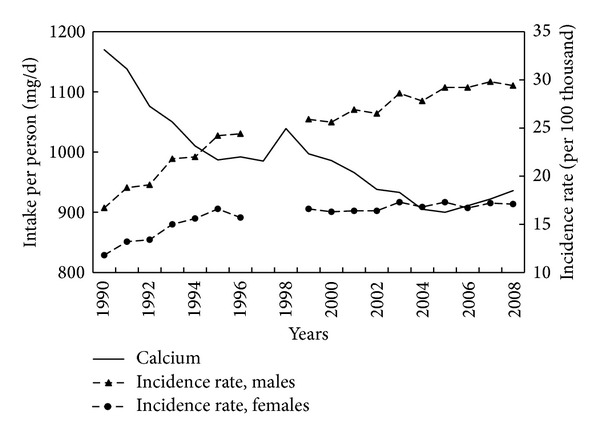
Calcium intake and colorectal cancer morbidity 1990–2008.

Data for calcium, a potentially protective factor, were analysed only since 1989 as in this year a constant decline in milk and dairy products consumption was initiated. Between 1990 and 2004, intakes declined by almost one quarter (Figure 9). A strong negative correlation was observed between calcium intake and incidence rates in the period 1990–2008 (−0.92 for men and −0.90 for women; *P* < 0.001).

**Figure 10:**
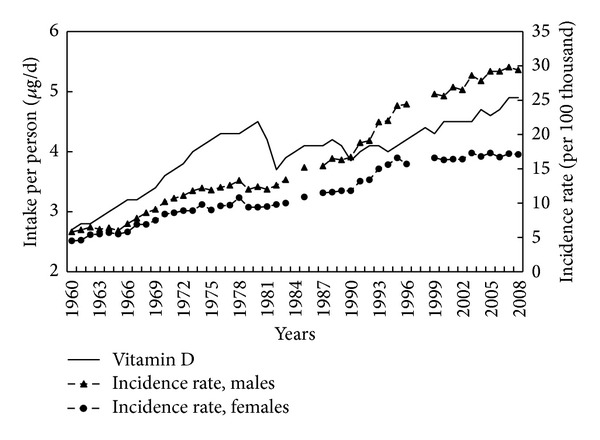
Vitamin D intake and colorectal cancer morbidity 1960–2008.

Between 1960 and 2008, intake increased (Figure 10), the apparent drop between 1980 and 1982 being due to the economic crisis which was associated with a decline in meat and milk consumption. Correlation analysis over the whole period did not show any significant association, but this may be due in part to the break in continuity of consumption over this period. The steady increase after 1990 could, however, be said to be associated with the corresponding rise in incidence (0.96 for men and 0.83 for women; *P* < 0.001).

**Figure 11:**
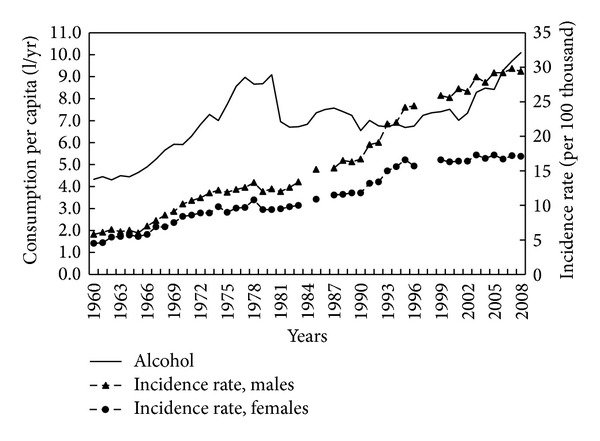
Alcohol consumption and colorectal cancer morbidity 1960–2008.

Apart from diet, colorectal cancer development may also be related to alcohol consumption and tobacco smoking. Reported alcohol consumption between 1960 and 1980 appeared to virtually double (Figure 11). There was then a plateau in the 1980s and 1990s. This was followed by a sharp rise since 2000. The correlation between alcohol consumption and colorectal cancer over the entire period 1960–2008 was moderate for both men (*r* = 0.68, *P* < 0.001) and women (*r* = 0.66, *P* < 0.001).

**Figure 12:**
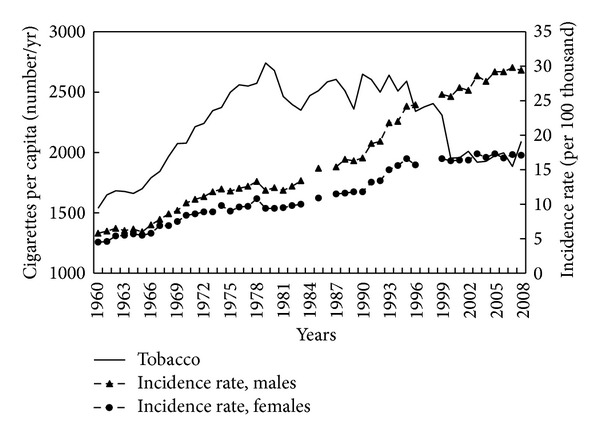
Tobacco smoking and colorectal cancer morbidity 1960–2008.

In the 1960s and 1970s, the number of cigarettes smoked per capita rose steadily, and into the mid 1990's remained high (Figure 12). After 1995 there was an evident decline, and in recent years the number of cigarettes smoked has reached plateau not far from the values in the 1960s. In the earlier period (1960–1989), the correlation between tobacco use and CRC was strong for both men (*r* = 0.85, *P* < 0.001) and women (*r* = 0.83, *P* < 0.001).

## 4. Discussion

Two subperiods may be distinguished in the analysis of CRC incidence in Poland [2, 3]. The first one, from 1960 to 1995, is characterised by a steady increase in rate, and the second, from 1996 to 2008, showed a plateau in morbidity in women, and a continuing (albeit slower) increase in men. We assume that this phenomenon is most probably connected with the specific observed changes in dietary patterns, alcohol consumption, and tobacco smoking in the Polish population, although of course other factors (discussed below) may also have contributed.

Overweight and obesity, especially abdominal fatness, are conducive to colorectal cancer incidence [1, 24]. Studies in the last decade suggest that over a half of adult Poles are overweight or obese, and very often this obesity is abdominal [25, 26]. Unfortunately, no long-term, national, systematically repeated anthropometric studies have been carried out in Poland, so it has not been possible to assess statistical correlation of body size and cancer incidence. It may be that consumption of fats and sugar, which are likely to contribute to high levels of overweight and obesity, could be interpreted as markers of excessive body weight.

Excessive body weight is a risk factor for the majority of malignant cancers, although colorectal cancer belongs to the group of tumours with a particularly strong relationship between raised BMI and increased risk of death [27]. The mechanism of the relationship between overweight and a higher risk of tumour development remains unclear. The key role is most probably played by hyperinsulinemia and insulin resistance as well as an increase of bioactive IGF-1. Both insulin and IGF-1 affect the increase in abnormal cell proliferation and reduction of apoptosis [4]. It is assumed that the excessive energy intake during childhood is conducive to the development of larger organs with a larger number of cells [28]. However, excessive energy intake during adulthood contributes to the intensified division of mucous cell membrane. Numerous cohort and population studies have observed a positive correlation between the CRC incidence and overweight and obesity. Based on the studies subjected to meta-analyses, it has been also estimated that the percentage of colon cancer cases caused by excess body weight amounts to 11% for women and men [24, 29, 30].

High fat consumption, especially animal fats, might also have a direct impact on the development of cancerous lesions. Higher intakes of animal fats leads to an increased production of prostaglandins (e.g., PGF2) which induce development of tumour cell clones (promotion stage) and promote further development of cancer (progression stage) [31, 32]. These studies suggest that high fat consumption may also increase colorectal cancer risk by increasing sterols production and changing the composition and activity of intestinal bacterial flora. In our study, the consumption of both total fats and animal fats may have had an impact on CRC incidence, in particular during the phase of the most rapid increase in incidence. However, the results of surveys exploring relationships between fat intake and CRC risk are controversial. For example, a cross-sectional study in a Portuguese population demonstrated increased colorectal cancer risk regarding the lowest versus the highest intake for total fat, saturated fat, and cholesterol [33]. On the other hand, data from the Fukuoka Colorectal Cancer Study, a population-based case-control study in Japan indicated that the intake of total fat, saturated fat, or n-6 PUFA showed no clear association with the overall or subsite-specific colorectal cancer risk [34]. There was a strong inverse association between n-3 PUFA and CRC risk.

The mechanism whereby high red meat consumption increases colorectal cancer risk is not precisely specified, even when high fat intake associated with red meat consumption is taken into consideration [35]. The method of food preparation itself, with all probability, may impact on the process of carcinogenesis. It is assumed that some specific carcinogens, especially heterocyclic amines, may be produced in the process of prolonged meat grilling or frying in high temperatures [1]. These compounds may interact with certain genes responsible for specific proteins (APC and *ras* protein) production. The mutated *ras* protein (which occurs in approximately 40% of CRC cases) contributes to a situation where the cell is constantly ready to divide, whereas the APC protein leads to disorders in the regulation of cell division frequency.

Contrary to expectation, there was a weak inverse correlation between the red meat consumption and CRC incidence in Poland since 1975. In the early 1990s, red meat consumption was very high (of the order of 60 kg/person/year), which could have had an effect on the increase of CRC incidence. The subsequent decrease in consumption of products from this group and its stabilisation at a lower level than in previous years could be one of the causes for the lack of increase in the CRC incidence among women and decreased rate of increase among men. Also, the type of meat consumed (a shift toward fresh or frozen meat and away from preserved meats) may also have been influence onincidence trends (slower increase) after 1996.

The fact that incidence rates reached a plateau in women and showed a reduced rate of increase in men could be related, *inter alia*, to this marked decline in red meat consumption. Results of studies on individual diets show that the average meat consumption among women is much lower than among men (142 g versus 260 g a day) and show differences in the types of meat products consumed [36]. The share of red meat in the diet of women is 56% and in the diet of men as much as 70%.

Much attention was devoted to investigating the role of fibre in colorectal cancer etiopathogenesis [31]. Some studies show that high fibre intake can significantly reduce the risk of adenoma and cancer in this part of the digestive tract [37, 38]. It is probable that fibre increases stool volume and decreases transit time. It is thus conducive to reducing the concentration of carcinogenic substances in the stool and shortening the time of their interaction with the mucous membrane. Also, certain fibre fractions (such as lignins) absorb on their surface large volumes of bile acids whose metabolites (secondary bile acids) are considered carcinogenic. Fermentation of soluble fibres and undigested starch, which results in decreasing stool pH, can also play a protective role as well as reduce the rate at which primary bile acids are converted into secondary bile acids that are known to be more toxic [39]. Bacterial fermentation also produces short chain fatty acids which can play an anticarcinogenic role, as they induce apoptosis which fosters the elimination of mutated cells [1, 40]. Also, prebiotics in dietary fibre, such as inulin and oligofructose, stimulate the growth of endogenous bacteria (*Lactobacillus*, *Bifidobacterium*), which again can help to inhibit carcinogenesis [41, 42].

The association between diet and CRC has been studied for many decades, with equivocal results. In the EPIC study dietary fibre in food was inversely related to incidence of large bowel cancer [43]. According to the conclusions from EPIC study, in populations with low fibre intake, an approximate doubling of total fibre intake from food could reduce colorectal cancer risk by 40%. Results of the Multiethnic Cohort Study conducted in Hawaii indicated that fibre was inversely associated with CRC risk in men but not in women [44]. The Japan Collaborative Cohort Study supported potential protective effects of dietary fibre against colorectal cancer, mainly against colon cancer [45]. The decrease in CRC risk with increasing dietary fibre intake was larger in men than in women.

However, a few studies have not demonstrated a preventive role for dietary fibre on colorectal cancer occurrence. For example, in a large prospective cohort study conducted in 6 US states and 2 metropolitan areas, total fibre intake was not associated with CRC risk, whereas whole-grain consumption was associated with a modest reduced risk [46]. Moreover, results from the Shanghai Women's Health Study demonstrated no apparent associations for fibre intake with colorectal cancer risk [47]. There is also the paradox of high and rising CRC rates in Australia, despite population-wide intakes of fibre especially of cereal origin [48].

One hypothesis suggests that dietary fibre's anticarcinogenic effect may depend on its source. For example, studies conducted by the National Cancer Institute in the United States show that fibre contained in whole grain cereal products and fruit significantly reduced the risk of colon adenoma, while fibre contained in vegetables and legumes did not have this effect [49].

Also fibre components may play an important role. Fung et al. [48] suggest that the focus on dietary fibre may have been misdirected. Resistant starch, rather than nonstarch polysaccharides, may be a key protector of CRC.

O'Keefe et al. [50] suggest that CRC risk is determined by interactions between the external (dietary) and internal (bacterial) environments. The CRC incidence is dramatically higher in African Americans than in Native Africans; however fiber intake is the same. According to authors higher CRC risk in African Americans than in Native Africans is associated with higher dietary intakes of animal products and higher colonic populations of potentially toxic hydrogen and secondary bile-salt-producing bacteria.

Studies in Poland have shown a clear correlation between changes in fibre intake and CRC incidence rates. Cereals are main source of fibre in Poles' diet [18]. This is consistent with the above hypothesis that cereal fibre may reduce colorectal cancer risk.

The possible protective role of vegetables and fruit seems less clear-cut. Some studies have shown a protective role for total fruit and vegetables [51] (although Nomura et al. [52] showed protective effects only in men), sometimes only very weak effects [53], and sometimes stronger for distal than proximal cancers of the gut [54]. In Poland, there was no association of CRC incidence with total vegetable and fruit consumption, but this may be explained in part because of high average total levels of consumption compared with other parts of Europe [53, 55]. For total vegetable consumption there was a negative association (consistent with expectations), but a positive association (contrary to expectation) for fruit consumption, accounted largely by the increase in fruit consumption between 1990 and 2001 during a period of increase in CRC incidence in both men and women. Whether the rise in fruit consumption helped to slow the rate of increase in CRC incidence due to other causes cannot be assessed.

In recent years, emphasis has been placed on the significance of folic acid deficiency in the diet to development of malignant cancer [56–59]. Folic acid is necessary for methylation, including DNA methylation. DNA methylation results in reduced gene expression and methylation disorders may result in loss of control of protooncogenes. Epidemiological and clinical studies have shown that low folate content in the diet or in serum is associated with higher cancer and adenoma incidence, including colorectal adenoma, although in the case of adenoma the relationship was less clear [60]. In some instances, however, the preventive effect of folic acid in carcinogenesis has not been proven [61, 62]. Indeed, the study of Van Guelpen et al. [63] suggests that low folate concentration in blood may even mitigate colorectal cancer risk. Presumably both deficiency and excess of folic acid may affect different stages of carcinogenesis of the large intestine. With the correct large intestine mucous membrane condition, folate deficiency may be one of the factors initiating carcinogenesis, and their high concentration may protect against carcinogenesis. At the same time, low folate concentrations may reduce cancer progression, and high concentrations accelerate it. Overall, despite the controversy, it can be assumed that gradually declining levels of dietary folate in Poland were one of the reasons behind the increase in CRC rates observed over the years.

Evidence suggests that higher levels of calcium mitigate colorectal cancer risk [31]. It may prevent the carcinogenic effect of bile acids by creating insoluble salts, or it may directly affect carcinogenesis by decelerating cell proliferation. Baron et al. [64] have shown that administration of 3 g of calcium carbonate per day reduces the risk of colorectal adenoma recurrence in patients who had an adenoma removed. A Japanese study confirmed the beneficial effect of increased calcium intake and insoluble dietary fibre fractions on colon cancer risk [65].

In Poland, the calcium content in an average diet is relatively low. Of particular significance is the prolonged decline in intake between 1990 and 2005. This trend correlated inversely with CRC incidence after 1990, potentially reducing the impact of other favourable changes in the dietary pattern which took place over the same period.

Alcohol consumption increases CRC risk [13, 14]. Increased neoplasia may be due to direct genotoxic effects, production of reactive oxygen species, and interference with folate metabolism. A review of 16 cohort studies found that there was 1.5–1.63 elevated CRC risk in those in the highest compared to the lowest alcohol intake category [66], with a clear dose-response relationship showing a 15% increase in CRC risk with each 100 g per week of alcohol consumed. There were no differences in risk according to the type of alcohol. Data on alcohol consumption in Poland suggest that it may be one of the factors contributing to high colorectal cancer incidence. In the 1980s and 1990s alcohol consumption was stabilized. This is explained in part by rationing and an attempt to limit illegal production but may also represent a degree of underestimation [67]. So it is possible that the real influence of alcohol consumption on CRC incidence could be higher than correlation coefficients indicate.

Apart from the factors related to diet and alcohol consumption, tobacco smoking may also have had an impact on colorectal cancer incidence. Tobacco smoking produces a variety of genotoxic agents such as heterogeneous nitrosamines and polynuclear aromatic hydrocarbons [12]. It is therefore not surprising that cigarette smoking is associated with an increased incidence of a variety of cancers including colon and rectum. In our studies, the increasing number of cigarettes smoked on average in Poland was clearly correlated with CRC incidence in the years 1960–1989. These variables were not correlated in the later years, but it seems that the reduction in tobacco use after 1995 could, along with other factors, account in part for the lack of increase in the CRC incidence rate among women and the reduced rate of increase among men.

The analyses above show that the increase in CRC incidence in the years 1960–1995 could be the simultaneous effect of a number of unfavourable dietary factors, such as an increase in edible fats consumption, particularly animal fats; an increase in sugar consumption; high consumption of red meat; and a decrease in consumption of products rich in dietary fibre and folate. The latter factors may have acted indirectly, mediated through an increase in the prevalence of overweight and obesity in the Polish population.

From 1998, the incidence in women stopped increasing, and the rate of increase amongst men slowed down. Again, these changes were probably due to overlapping effects of promoting and inhibiting factors. Consumption of alcohol and total edible fats increased while consumption of products rich in calcium decreased, creating favourable conditions for colorectal cancer. At the same time, consumption of red meat, animal fats and sugar decreased, accompanied by an increase in vitamin D intake and a decrease in the number of cigarettes smoked. Again, these factors can be assumed to have contributed to the more favourable trends in CRC incidence trends after 1995. The positive effect might have been greater if not for the decrease in the consumption of products containing fibre and folate.

*Other Factors.* A number of other factors may have influenced colorectal cancer rates in Poland over the time period under investigation. For example, they may include some ecological factors (pollution) and those related to life style (low physical activity, stress).

Moreover, the reason for increasing incidence rates is an ageing population [15].

Also the changes in diagnostic procedures, improved medical equipment and better opportunity of screening could influence the incidence rates.

*Limitations of the Study.* There are several potentially important limitations relating to the reported findings. First, the ICD went through four revisions between 1960 and 2008 (7th edition to 10th edition) [68]. While the alignment between editions has been carefully scrutinized for the purposes of this analysis, there will no doubt be variations in actual disease classification that will have taken place over time. Given the size of the change in incidence rates, however, this is unlikely to have had a major impact on the overall conclusions.

Second, the quality and comprehensiveness of data in the Regional Registries in Poland differ significantly [15]. According to assessments by independent experts, cancer causes are “under notified.”

Third, the population averages for consumption do not give separate values for men and women nor consumption at the individual level. It must be assumed that there are some differences between the changes in rates of consumption by sex, and these may in part account for some of the observed differences in the apparent associations observed. That said, many of the correlations are equally strong for men and women, suggesting that differences in the changes in the levels of consumption between sexes may be small in relation to the extent of changes in consumption observed over time.

Moreover, estimates of nutrient intakes based on national food availability data and food composition tables may not reflect actual levels of consumption due to preparation losses, food wastage, and cooking losses of volatile nutrients such as folate [69]. Alcohol consumption is also notoriously difficult to estimate as is tobacco use (although both can to a degree be verified against tax revenues).

There are no data on fibre components in the Polish food composition tables. So it is impossible to analyse the impact of selected components on CRC risk.

Finally, the correlations are limited in two ways. They do not take into account lag time between the changes in diet and incidence rates. Previous attempts to take lag time into account have not always been convincing [70]. Conducting the correlation analyses without attempting to take lag time into account may not, therefore, have a substantial impact on the overall conclusions, given the weaknesses inherent in any ecological analyses of diet-disease relationships. The other limitation of the ecological approach based on nonparametric correlations is its inability to take multiple factors into account through a multiple regression or partial correlation analysis, as the association between factors is not known and nonparametric approaches to partial correlation are much less robust than their parametric counterparts.

## 5. Conclusions

We have assumed that the dynamics of colorectal cancer incidence in Poland in the years 1960–2008 were a result of interactions between factors increasing and decreasing CRC risk. Analysis of these factors shows consistency with other international reported associations, but there are clearly some findings specific to Poland. This suggests that measures designed to improve the diet of the Polish population, as well as reducing tobacco smoking and alcohol consumption, could significantly reduce colorectal cancer incidence. However, an important question is whether dietary interventions are effective in lowering colorectal cancer risk. Only long-term studies covering several decades, examining how dietary pattern changes affect CRC incidence rates, will provide an objective assessment.

## Figures and Tables

**Table 1:** Correlations between dietary factors, alcohol consumption, tobacco smoking, and colorectal cancer morbidity rates, Poland, 1960–2008, by sex.

Factor	Dates	Men r*	Women r*
Edible fats	1960–2008	0.93	0.92
Animal fats	1960–1989	0.86	0.84
Red meat	1960–2008	−0.41	−0.41
Sugar	1960–1989	0.88	0.89
Dietary fibre	1960–2008	−0.92	−0.92
Vegetables	1990–2008	−0.66	−0.67
Fruit	1990–2008	0.70	0.62
Folate	1960–2008	−0.80	−0.80
Calcium	1990–2008	−0.92	−0.90
Vitamin D	1990–2008	0.96	0.83
Alcohol	1960–2008	0.68	0.66
Tobacco	1960–1989	0.85	0.83

r*: Spearman rank correlation coefficient. All values, *P* < 0.001 except vegetables and fruit (*P* < 0.01).
